# An ethnography of mealtime care for people living with dementia in care homes

**DOI:** 10.1177/14713012241234160

**Published:** 2024-02-21

**Authors:** James Faraday, Clare Abley, Joanne M Patterson, Catherine Exley

**Affiliations:** Population Health Sciences Institute, 5994 Newcastle University, UK; Adult Speech and Language Therapy, The Newcastle upon Tyne Hospitals NHS Foundation Trust, UK; Population Health Sciences Institute, 5994 Newcastle University, UK; School of Health Sciences, 4591 The University of Liverpool, UK; Faculty of Medical Sciences, 5994 Newcastle University, UK

**Keywords:** dementia, meals, long-term care, caregivers, ethnography

## Abstract

Many people living with dementia have difficulties at mealtimes, which can result in serious complications for physical and mental health, leading to hospital admissions and even death. However, current training in mealtime care for staff working with this population has been found to be poorly reported, with variable effectiveness. It is essential that care home staff are able to provide good care at mealtimes. This study used ethnography to explore current practice in mealtime care for this population, identify good practice, and understand the factors influencing mealtime care. Approximately 28 h of mealtime observations were conducted in two UK care homes with diverse characteristics. Observations focused on interactions between care staff and residents living with dementia. Twenty-five semi-structured interviews were carried out with care home staff, family carers, and visiting health and social care professionals, to explore mealtime care from their perspectives. A constant comparative approach was taken, to probe emergent findings and explore topics in greater depth. Key thematic categories were identified, including: tensions in mealtime care; the symbolic nature of mealtime care; navigating tensions via a person-centred approach; contextual constraints on mealtime care; and teamwork in mealtime care. The findings indicated that a person-centred approach helps carers to find the right balance between apparently competing priorities, and teamwork is instrumental in overcoming contextual constraints. This evidence has contributed to development of a training intervention for care home staff. Future research should investigate the feasibility of mealtime care training in care homes.

## Introduction

People living with dementia are at risk of eating and drinking problems of various kinds, including dysphagia (Abdelhamid et al., 2016), difficulty recognising food and drink (Amella, 2002), and problems using cutlery (Social Care Institute for Excellence, 2015). Mealtimes can also be impacted by changes in a person’s behaviour (such as agitation and restlessness), and changes in relationship with carers (Porter et al., 2021). Such difficulties have serious consequences, including malnutrition, dehydration and aspiration pneumonia (Abbott et al., 2013; F. Bunn et al., 2016). Mental health and social well-being may also be impacted: eating and drinking are fundamental human activities with significant social and emotional associations (Burges Watson et al., 2018), and mealtimes have an important psychosocial aspect (Fetherstonhaugh et al., 2019; Keller, 2016). Thus, difficulties at mealtimes can be distressing for people with dementia, and those who provide their care (Egan et al., 2020).

Mealtime difficulties are prevalent among people with dementia in care homes (Alzheimer’s Disease International, 2014). Care home staff are responsible for facilitating safe, adequate and enjoyable oral intake (Skills for Care, 2015). Staff may do this by providing physical assistance with eating/drinking (Abdelhamid et al., 2016; Mann et al., 2019), prompting and supervising mealtimes and responding to signs of dysphagia to minimise risk of adverse effects (such as aspiration and choking). Activity of this kind is referred to in the literature as mealtime care (Liu & Kim, 2021; Whear et al., 2014).

Good mealtime care for this population can improve quality of life (Evans et al., 2009), provide reassurance for families (Alzheimer’s Society, 2013; Hanson et al., 2013) and may reduce hospital admissions (Richardson, 2015). However, evidence has shown the quality of mealtime care is variable (Lea et al., 2017; Milte et al., 2017), and there are limitations in staff knowledge of nutrition and hydration needs (Beattie et al., 2014). Moreover, there is a paucity of adequate training on mealtime care for people with dementia reported in the literature (D. K. Bunn et al., 2016; Fetherstonhaugh et al., 2019), and a need for more rigorous research on this topic (Egan et al., 2020; Faraday et al., 2019; Kelly et al., 2015; Morley et al., 2014; Pagnamenta et al., 2022).

Several recent studies have examined mealtime care for people living with dementia. For example, Douglas et al. (2021) identified barriers and facilitators for care home assistants providing mealtime care to residents with dementia, focusing on the individual and interpersonal level rather than wider contextual factors. Liu et al. (2020) investigated influencing factors on mealtime care across multiple levels including resident, caregiver, environment and policy, but took a broad view encompassing not only care homes but also the acute in-patient setting. Through an ethnographic study, Driessen and Ibáñez Martín (2020) looked closely at the issue of attending to differences between residents at mealtimes, emphasising the importance of knowing residents and tailoring care.

This study aims to build on previous research by exploring current practice in mealtime care for people with dementia who live in care homes, identifying good practice, and understanding the factors which influence mealtime care. The focus of the study is both the individual/interpersonal level (carer-resident interactions) and the broader organisational level. The context of the study is specific to care homes. The scope of the study is all aspects of mealtime care. The study was part of a larger intervention-development study to improve mealtime care for residents with dementia, which was also informed by evidence from a systematic review (Faraday et al., 2021).

## Methods

Ethnography was the chosen method for this study, since it enabled exploration of the culture, perspectives and practices in care homes, to generate rich and detailed accounts (Reeves et al., 2008; Timmermans & Tavory, 2012). (The term care home is used here to denote a long-term care setting registered to provide residential care and/or nursing care.) This study received ethical approval from the Social Care Research Ethics Committee (reference 19/IEC08/0020, 5th June 2019).

### Patient and public involvement (PPI)

A PPI advisory group of was established at the beginning of the study, comprising four family carers with direct experience of caring for people with dementia at mealtimes (one of whom had also worked in a care home). The group met four times during the study, to discuss issues including ethical approval, engagement with care homes, fieldwork methods, and accessibility for participants.

### Sampling and recruitment

Maximum-variation purposive sampling (Palinkas et al., 2015) identified care homes for the study from a list of care homes registered for dementia care in the local area, obtained via the Care Quality Commission (CQC) website. To select the homes, this list was separated into large homes (50 or more beds), and small homes (fewer than 50 beds), and these homes were then ordered so that, when juxtaposed, each pair of homes differed in at least one other characteristic (such as size and scope of organisation). This enabled investigation of mealtime care in care homes that differed in certain respects to see if those differences impacted on care. Managers of the top pair of care homes were contacted in writing, to outline the study and invite their participation. This was followed up by telephone contact and individual face-to-face meetings, where both managers gave verbal agreement for their care homes to take part in the study. They subsequently provided written confirmation of this, and then a period of orientation took place in each home, during which recruitment began.

There were four participant-types in the study: care home residents, care home staff, family carers, and visiting health and social care professionals. Care home residents were eligible if they had a dementia diagnosis in their care records. The approach to consent was developed in consultation with the PPI advisory group. Senior care home staff made the initial approach to eligible residents, providing a study summary sheet and asking if they would like to meet the lead researcher (JF) to find out more about the study. If so, during this meeting JF assessed whether they had capacity to make a decision about consenting, following guidelines of the Mental Capacity Act (MCA) Code of Practice (2007). For residents lacking capacity, staff identified a personal consultee (Evans et al., 2020), contacting them to ask their opinion about whether the person would wish to be involved in the project. Other participant-types were approached by JF to seek consent.

### Study setting and participants

Observations and semi-structured interviews took place in two care homes in northeast England. Home1 had between 30 and 40 beds, and was part of a large national chain of care homes. Home2 had nearly 100 beds, and was part of a small, local group of care homes.

Eighty-seven people were recruited to participate in observations in Home1 (*n* = 30) and Home2 (*n* = 57), comprising 27 residents, 51 members of care home staff, seven family carers and two visiting health and social care professionals. There were 13 h of observations in Home1, and 15 h in Home2, over a time-period of 4 months, from September to December 2019.

Twenty-five people took part in interviews: 11 in Home1; 14 in Home2. Purposive sampling was used to explore the views and experiences of a broad range of participants (Patton, 2002). Across the two homes, those taking part in interviews were 17 members of care home staff, six family carers, and two visiting health and social care professionals. A more detailed breakdown of interview participants is provided in Table 1. Each interviewee took part in one interview. Twelve people from Home1 declined to take part in the study, as did seven people in Home2. A common reason given, particularly by staff, was lack of time.

**Table 1.** Interview participant types.

Participant type	Number	
Care home staff	Home1	Home2
Care assistants	3	5
Senior carers/Nursing staff		3
Management staff		2
Kitchen staff	1	3
Family carers	5	1
Visiting health and social care professionals	2	

### Data collection and analysis

Data collection was conducted by JF, and comprised observations (including informal conversations), and semi-structured interviews. Observations took place at breakfast, lunchtime and teatime, as well as other times of day. Locations included dining rooms, lounge areas and bedrooms (but only if acceptable to the resident). The focus was on interactions between participating residents and staff. Sometimes, participating family carers were also present and directly involved in mealtime care. Moderate participant observation was used (Spradley, 1980), with the researcher present, identifiable, and interacting to some extent with the people being observed. Field-notes were written discretely in situ, or elsewhere as soon as possible after the observation. The researcher either sat next to residents, or at a distance, and was guided in this by staff, and the residents themselves. Semi-structured interviews (audio-recorded and transcribed) were used to explore the views of care home staff, family carers, and visiting health and social care professionals, particularly on what helps and hinders people with dementia to have a good experience at mealtimes. Topic guides were developed in consultation with colleagues and informed by relevant literature. The topic guides covered areas including residents’ experience of mealtimes, supporting people with dementia at mealtimes, and training on mealtime care (content and delivery). They were used to guide discussion in interviews, rather than to strictly prescribe it (Ritchie et al., 2013). Informal conversations were employed to elicit residents’ views.

Data collection and analysis followed the principles of Constructivist Grounded Theory (Charmaz, 2006). A constant comparative approach was taken to probe emergent findings and explore topics in greater depth. The discovery of early patterns and themes allowed data collection to be refined. For analysis, observation field-notes and interview transcripts were printed and annotated with initial codes, focused codes and memos. Annotations were done by hand, with initial codes and focused codes written in the margins, using actions words where possible. Codes and memos were refined, conflated, added to and deleted, then manually clustered together using Microsoft software, to create categories and sub-categories which sought to answer the broad research questions specified by the study. Flip-chart paper was then used to sketch out connections between codes and categories, making decisions about which of these fitted together, and which were sufficiently strong and coherent to stand alone. Textual descriptions of categories and sub-categories were written, expanding on existing memos and adding new ones. Data extracts were chosen to illustrate categories and sub-categories.

Theoretical sampling (Charmaz, 2006) determined the point of data saturation, with preliminary ideas examined via further empirical enquiry. This iterative process was interrupted by the COVID-19 pandemic, which meant data collection paused for several months. When research activity resumed, categories were refined through conversation with key participants (Albas & Albas, 1988, 1993), via telephone interviews.

### Trustworthiness of research

Various steps were taken to ensure trustworthiness of the research. There were regular discussions about data, codes and categories with the PPI advisory group and between all authors, to develop, refine and test the analysis. In addition, reflexive notes were written during data collection and analysis (Davies et al., 2004; Watt, 2007). The lead researcher (a Speech and Language Therapist by background) was careful to acknowledge and reflect on previous experiences from clinical work, to reduce risk of professional bias. Finally, the proposed theoretical categories were tested against relevant literature (Dey, 2012; Dick, 2012).

## Findings

Key thematic categories are described and exemplified below. The categories are: tensions in mealtime care; the symbolic nature of mealtime care; navigating tensions via a person-centred approach; contextual constraints on mealtime care; teamwork in mealtime care. These categories provided an understanding of current and good practice in mealtime care, and the factors which influenced this. To preserve participants’ anonymity, pseudonyms are used for residents, and descriptive alphanumeric labels are used for other participants. For example: Staff1_10 stands for the 10th member of staff to be recruited in Home1.

### Tensions in mealtime care

Mealtime care was focused on various priorities: promoting choice, independence, social well-being, and adequate nutrition/hydration. However, these priorities sometimes seemed to be in tension with one another. One such tension existed between independence and adequate nutrition/hydration. Some residents were able to eat more if directly assisted:

Gracie tries to eat the pineapple upside-down cake, but has difficulty with maintaining the food on the spoon. So Staff1_8 helps her by cutting up the pudding into smaller pieces and placing a piece onto the middle of her spoon. Shortly afterwards Staff1_8 steps in again, takes the spoon, and begins to directly assist Gracie. Gracie looks unhappy about this initially, but then accepts some spoonfuls. [Field-notes_dining-room_Home1_lunchtime_01/11/19]

There was also tension between choice and social well-being. Some residents preferred to eat in their rooms, not communal areas. There were differing views on this from care staff:

“If they’re in their own room it’s because they want to be on their own. Because the mealtimes can get really loud, and for some residents it’s really annoying. They just want to be on their own, so we do give them that opportunity if they want.” [Interview_Staff2_38]

“There’s certain residents that just don’t want to come out of their room … there’ll be times where you can persuade someone to come out of their room because obviously it’s much better when someone’s – it’s just more social, you know, when there’s more people in the dining room – so we promote that.” [Interview_Staff1_06]

The challenge lay in how much to encourage. A family member felt that staff did not go far enough in encouraging her mother to leave her bedroom:

“She needs to be encouraged more. I don’t feel as if she’s encouraged enough to go and socialise. Now I’ve just asked her there, I says, ‘we’ll go along the other end and have a cuppa.’ Now she’ll do that because I’ll say, ‘Come on. Stand up.’ And I really firmly encourage her. The staff will say to me, ‘Well she doesn’t want to.’ Well no, she doesn’t want to because she doesn’t realise it’s isolating her, so you need to encourage her – find a strategy to use.” [Interview_FamilyCarer2_1]

The resident had expressed a preference to stay in her room for meals – but her daughter believed her best interests were served by encouraging her to leave. It was possible to conceptualise these kind of individual tensions as being, in broader terms, a tension between autonomy and care. Carers were faced with making frequent decisions about ‘what is best’ for residents. Cognitive impairment meant many residents lacked capacity to make informed decisions themselves. Challenges arose when a resident’s implicit or expressed preference was perceived by care staff, family or other healthcare professionals to be detrimental to their well-being.

“We’ve had somebody who insistently would only eat certain foods and again, we ensured that got incorporated into their diet so as long as it didn’t have an impact on a medical condition such as diabetes, then we would allow it within moderation.” [Interview_Staff2_41]

The name of the setting encapsulated this tension between autonomy and care. The residents lived in a care home. As such, they were under the care of staff – but they were also in their home. Staff regularly made the point in interviews that ‘this is the resident’s home,’ when they were emphasising the importance of resident autonomy:

“Because it’s their home at the end of the day. If they were at home and they made themselves something … and they thought (I’ve done it before): ‘I don’t really fancy that’, then they’re able to go and get something else, make something else. So why can’t they have that here?” [Interview_Staff2_42]

In reality, residents did not have those same freedoms they would have experienced in their own homes. For example, residents were not able to make themselves a cup of tea. This idea was explored further with a senior carer:

“Of course it would be lovely in an ideal world for everybody to have all of that independence and be able to do the things they want to do, but you’ve seen yourself, it can be quite hectic in that kitchen at a mealtime, and all it takes is a member of staff to turn around too quickly and we’ve got a resident scalded, and for me, that wouldn’t be something I would be comfortable with. So it’s not about taking away their rights as such … but protecting them and trying to make the best decision to make sure these kinds of things don’t happen.” [Interview_Staff2_39]

To paraphrase the senior carer: residents’ autonomy was important, and to be promoted – provided it did not jeopardise their health and well-being. Carers faced difficult dilemmas like these every day – whilst managing multiple other demands, in a busy, dynamic environment.

### The symbolic nature of mealtime care

Staff cared about residents, as well as caring for them. Moreover, this caring instinct was heightened at mealtimes. Care staff expressed a desire for residents to successfully eat and drink, and were frustrated or anxious when this did not happen:

“It’s very difficult, because obviously you can’t make somebody open their mouth if they don’t want to ... It’s one of those things: you want them to eat and you just try to encourage. You can’t do anything else, other than try to tempt them, encourage them. You know what I mean? Obviously, we do – we put the spoon to their lips, we’ll tell them what it is on their plate, we’ll try to put a spoon in their hand even.” [Interview_Staff1_3]

It was clear this care assistant felt a responsibility for ensuring residents ate and drank well. At times, this feeling of responsibility seemed to cross over into a feeling of burden. As the interview was drawing to a close, they were asked if there was anything else they would like to say about mealtimes.

“Well no, I just [sighs]… It’s always hard to get it right, I think because not one day is the same and us girls – if somebody has a bad day we always get upset and worry about it. Because one thing about caring is wanting somebody to eat well and drink well because, you know, it’s all part of you being well, isn’t it?” [Interview_Staff1_3]

In both care homes there was pressure to encourage residents to eat and drink well. It seemed this pressure could originate internally (i.e., from a care assistant’s own personal and professional duty of care), and/or externally (e.g., because of family expectations). A member of senior staff in Home2 recognised this in colleagues:

“Staff can take it personally. They take it as a problem and then of course what doesn’t help and exaggerates everything is the fact that families are, ‘Well, are you trying her with that? Are you trying him with this and that and the other?’ ‘We’ve got beans, he likes beans,’ and they’ll try, and it might work once, so they think that was the answer, that staff aren’t doing their job and so you can get suddenly a melt-down of confidence and then – it feels pressured and: ‘We must get him to eat!’” [Interview_Staff2_28]

Part of the challenge appeared to be determining what ‘eating and drinking well’ actually looked like. A visiting professional in Home1 implied that care assistants were at times caught in a conflict between reality and expectation:

“We know that a lot of patients with dementia, their appetite subsides so I think that’s sometimes quite difficult for the staff if people are refusing to eat and we’re trying to promote that they should be eating and drinking.” [Interview_VisitingProfessional1_1]

Pressure to encourage eating and drinking could create a narrative about residents which was negative and unhelpful. That is, reduced eating and drinking was sometimes equated to ‘challenging’ or ‘difficult’ behaviour:

“If there’s a resident that’s being quite difficult, like say if they’re on a food or fluid chart, and we need to get something down them specifically because they’re on that chart, and if they refuse to eat – then that puts us in a bad position as well.” [Interview_Staff2_38]

It was evident from these conversations that carers construed a key part of their role as ensuring adequate nutrition and hydration for residents. They wanted residents to eat and drink. Thus, occasions when residents stated (or demonstrated) they did not want to eat or drink seemed to epitomise the tension between autonomy and care. Residents were expressing their will, whilst staff were attempting to care for them in a fundamental way. This was difficult terrain for staff to navigate.

### Navigating tensions via a person-centred approach

A person-centred approach was potentially transformative to mealtime care – something that enhanced all aspects of the mealtime, and served as a guiding principle where there was tension or uncertainty. To take the case presented above, of the resident who preferred to stay in her bedroom for meals: the resident’s daughter was concerned that a preference to stay in her room at mealtimes was detrimental to her social well-being. The daughter proposed a solution which took account of her characteristics, habits and history:

“When my mam broke her hand and she wouldn’t come out of the room... You know, that’s fine, but then it got where she wasn’t coming out of her room at all so the social isolation was starting. And then they were saying, ‘Ah well we’ll bring her out for breakfast.’ Well, my mam’s not a morning person and they should know that by now. She’s been here a year and a half so she likes to take it slow and I said, ‘Well I would prefer when mam gets up just leave her in her dressing gown and give her breakfast in her room. Encourage her to come out at lunch time when she’s more alert.” [Interview_FamilyCarer2_1]

Knowing that the resident was “not a morning person,” and adapting care accordingly, seemed to be key to this; a way of respecting their individual preferences whilst addressing a more general idea of social well-being. It seemed that a perspective of this kind could potentially help care staff prioritise well, and make good decisions at mealtimes. This was also relevant when staff were seeking to promote independence whilst ensuring residents ate enough:

“The ones who can’t cut their meat, you would say, ‘Are you fine now?’ You don’t want to start saying ‘Well come here I’ll feed you’. You know, you read their care plans that they’re alright to eat on their own. But the ones that you know can’t eat, you’re not going to cut it up and say, ‘Look it’s cut into tiny little pieces, right you try that yourself’… You know for a fact they’re not going to eat it, so you put your plate-guard on the back of it, and the ones who struggle a little bit you would put the plate-guard on and that helps them a lot. But the ones that can’t eat, I wouldn’t be leaving them. I would sit with them until it’s all gone.” [Interview_Staff2_31]

Some residents were able to eat and drink independently, but in so doing would be at risk of spilling food or drink onto themselves, onto the table, or onto the floor. This meant staff needed to think about how they could maintain their independence, and their dignity:

“I don’t think somebody’s independence should be took away because they make a bit of a mess. We’ve got one lady in here, if you give her a big spoon she’ll make a mess, but if you think ahead a little bit and give her the smaller spoon she doesn’t make quite as much mess. You don’t just automatically think, ‘oh she’s making a mess, we’ll feed her.’ … You should always look for something else.” [Interview_Staff2_25].

A person-centred approach was also helpful when staff were caring for residents who preferred unhealthy foods or drinks. A carer in Home1 described a resident who would only eat food if accompanied by a particular fizzy drink. The carer was aware that this resident would otherwise be unable to meet their nutritional needs. In the carer’s words, the fizzy drink was an imperfect solution – but it was, nonetheless, a solution:

“There’s another gent who … it was getting to the point where he wouldn’t eat without having coca cola. … and it’s just a case of… ‘Right, there’s the coca cola…’ But it isn’t a perfect solution… but it is a solution, if he’s only going to eat when he’s got a drink of coke to go with it…” [Interview_Staff1_6]

Being person-centred here led to a pragmatic way forward. There was acknowledgement among staff that for some residents, consumption of calories was the critical factor, rather than a balanced diet. Thus, by focusing on the person as an individual – their history, capabilities, preferences and so on – staff were better able to find a way through challenges and dilemmas.

### Contextual constraints on mealtime care

All carer-resident mealtime interactions occurred within a complex system – the care home. This comprised many different departments and staff-groups, as well as external elements. Each of these influenced mealtime care in different ways. The role of the kitchen seemed a prime example of this. Kitchen staff were tasked with producing food to certain timescales. In both homes, food trolleys were delivered by kitchen staff at regular times, and collected again approximately 1 h later. It was expected that trolleys would be loaded and ready when kitchen staff returned. A care assistant in Home2 explained that the lunchtime food trolley was delivered at 1 pm and collected at 2 pm:

Researcher: “Do you mean that you have to be finished by 2 pm?”

StaffB38: “Yeah. It’s not a long time at all. Especially if the trolley is late because then you’re rushing around.”

Researcher: “I suppose I’m just thinking, so what happens at 2 pm…? Would it be a problem if it just sort of dragged on after, sort of 2:30 or whatever…?”

StaffB38: “I think it would only be a problem for people who were in the kitchen because they’ve got to come and get the trolleys, take them back down, clean them, wash the dishes and bring them back up.” [Interview_Staff2_38]

This kind of tight schedule had negative consequences for mealtime care:

“They [staff] forget it’s their [residents’] social time, and they can try and rush at times. You’ve probably witnessed it yourself, where plates getting taken away or a meal is not finished and desert is getting put down and that gets on my nerves. To the extent like ‘give them time!’. It’s a 12-hour shift. There’s no reason for that dining room to be emptied in 20 minutes. Let them have that bit of social time.” [Interview_Staff2_42]

Care assistants were also constrained by their relationship to senior management. This was seen in the context of staff sitting and eating with residents, a recommended practice in some guidelines (Bournemouth University, 2018). In observations, it was more usual for staff to wait until their breaks to eat – or to eat quickly ‘on-the-go’. In this extract, two care assistants demonstrated contrasting approaches:

Most residents in the dining room have finished their cereal now, and some have moved onto cooked breakfast. Staff2_4 asks Staff2_35 if she’s having some breakfast – Staff2_35 says “I’ve pinched a slice of bacon”. Staff2_35 and Staff2_37 stand in the kitchen eating bacon sandwiches, while Staff2_4 takes her cooked breakfast over to Clara and Daphne’s table and sits with them. [Field-notes: dining room, Home2, breakfast 05/12/19]

In a later discussion with one of the carers, the implication was not all senior staff were in favour of the practice (and practical challenges were evident):

As the residents are finishing their pudding, Staff2_4 sits down with a portion of chicken pie, on a table with one resident. As she eats, I comment on the fact that she is doing this – she tells me “This is the only floor you’re allowed to do it.” I say I saw it happen on another unit earlier in the week. She says “Let’s put it this way – it depends which seniors are on!” She tells me it is actually quite difficult, because she will often still need to get up to assist a resident. In fact while we are talking, a nearby resident asks for another yoghurt – she stands to go to the kitchen, but Staff2_35 has overheard and gets the yoghurt instead. [Field-notes: dining room, Home2, lunch 16/11/19]

On further exploration, it appeared there was general recognition of the value of staff sitting and eating with residents, but an absence of ‘top-down’ commitment to make it happen. Consequently, some staff did not feel empowered or encouraged to give care in this way.

In addition, mealtime care was compromised by perceived or real influence from head office – as exemplified in this extract, where a staff member discusses responding to residents’ food and drink preferences:

“From a company policy, if [residents] have got a certain type of preference then usually it’s the family members that would provide that preference. Because obviously that preference is outside of our menu. And then you go along the lines of – we’ve got to control everything that comes into the building ... I’ve got to label it. I’ve got to check it and things like that. … We do try and pander to their needs, but as a company they don’t necessarily recommend it because it’s added cost.” [Interview_Staff1_14]

Administrative and financial considerations, alongside other organisational constraints, impacted on the care that staff were able to provide. At times there was a risk of the needs of the individual becoming subservient to the needs of the organisation.

### Teamwork in mealtime care

The strength of connection between different elements of the care home system was central to the quality of mealtime care. When they came together – through collaboration, sharing and problem-solving – care was at its best. Most obviously, this happened when care assistants worked together well, sharing knowledge and concerns:

“We’re a team. It’s no good me knowing what’s good for that resident if I don’t pass it on to everybody else because she won’t get the same care when I’m not here if that’s the case. Everybody knows and we all follow it.” 110 [Interview_Staff2_33]

Co-operation between different departments within the homes seemed more sporadic, but in Home1 in particular, kitchen staff and care staff were well-connected. This was epitomised by a member of kitchen staff who spent time each day in the dining room, helping with the mealtime service:

“If I had to trade being on time all day or rushing a resident, I think I would prefer to be late. I’m not going to rush them. I’ve spent nearly an hour one day upstairs, it’s just when I’ve got back to the kitchen then obviously I’ve got to push myself a little bit to catch myself up. But you can’t rush them and I don’t care how long it takes as long as they’re getting something. So it is a lot of pressure … but obviously you don’t show that pressure because it’s important for the residents. You want them to be relaxed, you want them to sit, enjoy the food and if that takes 10 minutes, if it takes an hour, I’m not bothered.” [Interview_Staff1_14]

There were also instances of senior staff and care assistants working together effectively. This seemed to happen particularly when facing the challenge of caring for a resident who was eating and drinking very little. Staff groups came together around these difficult cases:

“It’s sometimes difficult, and maybe that’s where supervision is important, discussing about a certain client, you know, ‘Have you got problems with anybody?’ … It’s about having that discussion and saying, ‘Look what you’re doing is fine, you’re offering different types of food, you’re doing a good job’. … But it’s about not making it that individual’s responsibility, but sharing the responsibility.” [Interview_Staff2_28]

Good communication, with everyone playing their part, seemed key to managing these challenges. It was also evident that a collaborative way of working may help to reduce feelings of burden and pressure:

“Sometimes you can go to assist somebody and they won’t accept anything from you, but they’ll accept it from somebody else. And especially the newer members of staff take that personally, but it’s about supporting them and letting them know ‘it’s not just you’.” [Interview_Staff1_15]

In addition, the care team around the resident extended beyond the boundaries of the home – to become a ‘wider care team.’ There were important interactions between care home staff and external health and care workers – such as GPs, dietitians, SLTs, and community nurses. This was not a deferential relationship, where responsibility for care was delegated. Rather, it seemed collaborative and mutual – a joint-effort between care home staff and the external team to resolve the problem:

“[Speech and Language Therapists] are good to advise and help. We tell them what we think would be best for that resident. Obviously, we’ve known [the residents] longer, what they will eat, what they won’t eat, what sort of fluid intake they’ve got…” [Interview_Staff2_42]

He [the GP] says that GPs are only able to do one-off assessments, so really good information from staff makes him feel safe. He gives an example of a nurse he has worked with previously, who kept really good charts and weights. And he says he feels reassured when [this carer] gives him information about a resident. [Field-notes_Home1_19/11/19]

Finally, teamwork in mealtime care was expressed through involvement of family carers. In Home1, Ian’s family visited every Saturday lunchtime. He preferred to eat in his room, and needed much assistance – his family helped with this:

Various family members are present. Ian is sitting in a comfy armchair. A member of care home staff has brought the first course on a tray – vegetable soup. The family suggest that they assist, and invite me to stay during the meal. His daughter takes the soup; she is sitting slightly behind Ian on the bed. She is directly assisting him, at a suitable pace. Ian seems to enjoy the soup and eats all of it. [Field-notes_resident’s-room_Home1_lunchtime_21/09/19]

Family carers were able to complement or augment staffing at mealtimes, and had certain advantages when assisting in mealtime care:

“Family have that time and that relationship … Today there’s somebody upstairs, a member of their family has come in to give them lunch … I know families have commitments, people still work and not everybody can do that and it doesn’t have to be family. It might be an old friend…” [Interview_VisitingProfessiona1_1]

However, it was apparent there were complexities in family involvement:

“Some residents perhaps would be better and eat better and have a better diet when the family members are present at a mealtime. It calms them down and makes them feel more comfortable whereas others, it could be a big distraction having their family there and draw away from the fact that it’s mealtimes, so it is still very individual and that’s the balancing act within a care home.” [Interview_Staff2_39]

This was the same ‘balancing act,’ recognising and meeting the needs of individual residents in a group setting, that necessarily informed all aspects of mealtime care.

## Discussion

**Figure 1.**
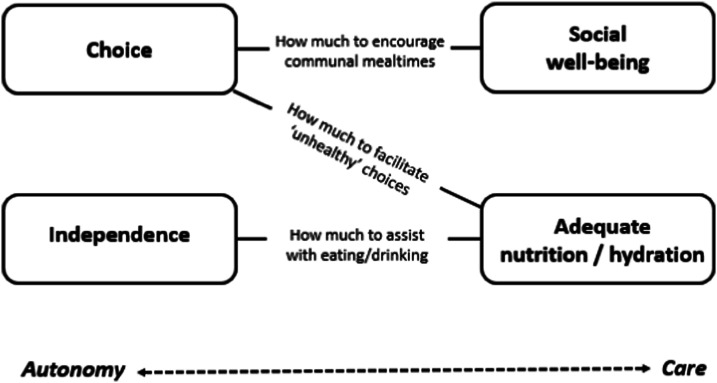
Examples of tensions in mealtime care.

This study contributes new knowledge about good practice in mealtime care for people with dementia living in care homes, and about contextual constraints on practice. It confirms the importance of a person-centred approach (see also Driessen & Ibáñez Martín, 2020; Murphy et al., 2017; Nell et al., 2016; Palese et al., 2018). Person-centredness is a well-known concept in health and social care, and is now considered integral to all aspects of dementia care (NICE, 2018). If the person is viewed holistically, and as an individual, care can be provided which meets their needs and priorities. This study develops this idea by showing that person-centred care enables carers to find a way through apparently competing priorities that arise at mealtimes (see Figure 1). When care staff took into account a resident’s preferences, capabilities and other personal factors, they often seemed able to navigate a path through tensions in mealtime care – meeting the resident’s care needs without compromising their personhood (Kitwood & Bredin, 1992). In particular, it is proposed that a person-centred approach is helpful in reconciling autonomy and care – an opposition also seen in the broader dementia care literature. Evans et al. (2018), for example, describe a paternalistic concern with resident safety potentially conflicting with advocacy for the individual as a free agent (p. 261). They note that the first theme tends to take precedence in practice, with care home staff erring on the side of caution. In our analysis of mealtime care, this seemed to depend on the perceived level of risk. If staff thought a resident’s preference to be both unwise and harmful – for example, wanting to have a lot of salt on meals – they tended to be paternalistic and restrictive in their response. If they thought a resident’s preference to be unwise but (relatively) harmless – for example, wanting to stay in their bedroom for meals – they were more likely to facilitate this.

**Figure 2.**
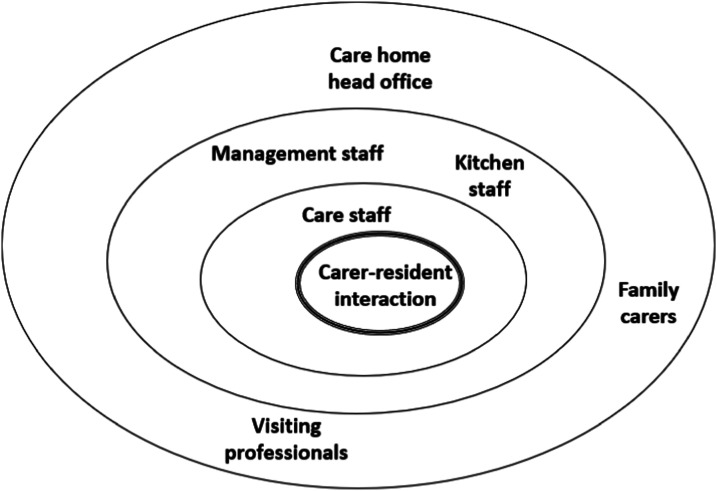
Contextual factors in mealtime care.

In addition, mealtime care operates within a complex system which can both constrain and enable good care (Peryer et al., 2022). Teamwork was seen to be a crucial mechanism for overcoming organisational constraints, tackling dilemmas and uncertainty, and reducing emotional burden. Effective teamwork is already known as an important component of long-term care in general, and dementia care in particular (Etherton-Beer et al., 2013; Gilster et al., 2018; Gordon et al., 2018). In the context of mealtimes, the value of teamwork has been identified by Shune and Linville (2019), who proposed the creation of interdisciplinary teams to oversee mealtime care – which they termed ‘dining teams.’ This study’s findings build on this by indicating that the mealtime care team should not be limited to health and social care staff. Rather, everyone involved in the life of a resident – inside and outside the care home – may come together at different times and in different combinations to improve mealtimes for that person: the ‘wider care team.’ Teamwork can also help defuse the emotional burden associated with mealtime care, and in particular with reduced eating and drinking (Featherstone et al., 2019; Hopkins, 2004). Finally, teamwork has a specific role in mitigating the constraints of time and resource that can otherwise hinder care, and has a positive effect on the complex system in which mealtime care operates (Figure 2), in a way that enhances care.

### Limitations

The care homes which participated in the study were different in size (number of beds) and ownership (national/regional/local). However, in other ways they were similar (e.g., both were located in socially-deprived urban areas). A third care home which agreed to take part was in a suburban area with less social deprivation, but no observations could be conducted there due to the onset of COVID-19 lockdown measures.

Participants were recruited from a variety of roles and professions, including care assistants, senior staff, healthcare professionals and family members. Amongst the residents, however, there was a certain group which was under-represented – those with advanced-stage dementia. Recruiting within this group proved to be challenging. These residents generally lacked capacity to consent to participation, and therefore consultee opinion was required. Although a relevant consultee was usually identified, in many cases they did not respond to contact. In addition, these residents were often in more compromised health than those whose dementia was less advanced. Understandably, the care home staff who helped with recruitment tended not to approach residents who were unwell, likely feeling this was not in their best interests. Thus, there were relatively few residents in the study whose dementia was very advanced. Findings should be interpreted in light of this, and future studies must consider carefully how to be more inclusive of this population. Finally, future studies should carefully consider the equity of data collection methods. To collect verbal data, this study used informal conversations with residents, and semi-structured interviews with other participants. It may be that using a common method of data collection across all participants would be preferable.

### Implications for practice and policy

The findings provide valuable principles informing both practice and policy in mealtime care for residents living with dementia. A person-centred approach, focusing on residents’ history, capabilities, preferences and prognosis, helps resolve tensions between competing priorities. Teamwork between care staff, kitchen staff, management, external health and care professionals, and family carers is key in overcoming contextual constraints. Enacting person-centred care at mealtimes may include staff having ready access to comprehensive, up-to-date information about residents, and ensuring relevant knowledge and insights about residents is well-communicated. Policy in this area should account for the complex system in which mealtime care operates, and the fact that it is best delivered in a collaborative way. All groups involved in mealtime care should be consulted and have a voice in the development and refinement of relevant policy. This would be facilitated by better inclusion on relevant committees and panels, for example in the creation of mealtime care guidelines. Structures and initiatives that promote greater connection within and beyond the home should be encouraged. This may include a regular forum for discussing mealtime care, or participation of the wider care team in training events.

## Conclusion

Mealtime care for people living with dementia in care homes is complex and challenging. This study has explored current practice in mealtime care, and identified good practice. The findings indicate that a person-centred approach helps carers to find the right balance between apparently competing priorities. Teamwork is instrumental in overcoming contextual constraints. This evidence has contributed to development of a training intervention for care home staff. Future research should investigate the feasibility of mealtime care training in care homes.
